# The growth and phosphorus acquisition of invasive plants *Rudbeckia laciniata* and *Solidago gigantea* are enhanced by arbuscular mycorrhizal fungi

**DOI:** 10.1007/s00572-016-0729-9

**Published:** 2016-08-31

**Authors:** Marta L. Majewska, Kaja Rola, Szymon Zubek

**Affiliations:** Institute of Botany, Faculty of Biology and Earth Sciences, Jagiellonian University, Kopernika 27, Kraków, 31-501 Poland

**Keywords:** Arbuscular mycorrhizal fungi (AMF), Arbuscular mycorrhiza (AM), Asteraceae, Giant goldenrod, Golden glow, Plant invasions

## Abstract

While a number of recent studies have revealed that arbuscular mycorrhizal fungi (AMF) can mediate invasive plant success, the influence of these symbionts on the most successful and high-impact invaders is largely unexplored. Two perennial herbs of this category of invasive plants, *Rudbeckia laciniata* and *Solidago gigantea* (Asteraceae), were thus tested in a pot experiment to determine whether AMF influence their growth, the concentration of phosphorus in biomass, and photosynthesis. The following treatments, including three common AMF species, were prepared on soils representative of two habitats that are frequently invaded by both plants, namely fallow and river valley: (1) control—soil without AMF, (2) *Rhizophagus irregularis*, (3) *Funneliformis mosseae*, and (4) *Claroideoglomus claroideum*. The invaders were strongly dependent on AMF for their growth. The mycorrhizal dependency of *R. laciniata* was 88 and 63 % and of *S. gigantea* 90 and 82 % for valley and fallow soils, respectively. The fungi also increased P concentration in their biomass. However, we found different effects of the fungal species in the stimulation of plant growth and P acquisition, with *R. irregularis* and *C. claroideum* being the most and least effective symbionts, respectively. None of AMF species had an impact on the photosynthetic performance indexes of both plants. Our findings indicate that AMF have a direct effect on the early stages of *R. laciniata* and *S. gigantea* growth. The magnitude of the response of both plant species to AMF was dependent on the fungal and soil identities. Therefore, the presence of particular AMF species in a site may determine the success of their invasion.

## Introduction

A high tolerance to environmental conditions, short life cycle, good seed viability, and dispersal mechanisms as well as strong allelopathic effects are listed among the capacities of invasive plant species that facilitate their expansion in new habitats (Kornaś 1996; Tokarska-Guzik et al. 2012). Recent studies suggest that soil microorganisms, particularly arbuscular mycorrhizal fungi (AMF), may also play an important role in the success of invasive plants (Pringle et al. 2009; Shah et al. 2009). This could be due to increased nutrient acquisition, growth, and protection against biotic (pathogens) and abiotic (drought) stresses of mycorrhizal plants (Smith and Read 2008, Shah et al. 2009). Several studies have focused on the impact of AMF on invasive plant species. The improvement in the growth and vegetative and reproductive attributes of *Anthemis cotula* upon AMF inoculation was reported by Shah et al. (2008a, b). Fumanal et al. (2006) showed a positive influence of AMF on the growth and development of *Ambrosia artemisiifolia*. It was also found that AMF promoted *Microstegium vimineum* biomass, increased P uptake, and altered plant morphology, increasing the number of stolons and aerial roots (Lee et al. 2014). *Centaurea stoebe* exhibited a positive growth response to AMF, either growing alone (Harner et al. 2010) or with some neighboring plants from which it can exploit resources via mycelia (Marler et al. 1999; Zabinski et al. 2002; Callaway et al. 2004; Carey et al. 2004). Bray et al. (2003) demonstrated that the relative growth rates and leaf area ratio of *Ardisia crenata* were higher for AMF-inoculated seedlings. The results reported by Smith et al. (2008) indicated that AMF are beneficial for the survival and growth of *Vincetoxicum rossicum* seedlings. Moreover, *Solidago canadensis* was found to change the species composition of AMF communities in soils and, as a consequence, had promoted the growth and abundance of AMF species that positively influenced its own growth (Zhang et al. 2010; Yang et al. 2014; Yuan et al. 2014). It was also suggested that AMF may enhance the competitive interactions of alien plants due to improved herbivore defensive mechanisms through changes in root exudates and shoot compounds (Shah et al. 2009). However, the nature of mycorrhizal association is variable, ranging from mutualistic to mildly parasitic, and depends upon the plant and AMF identities as well as environmental conditions (Smith and Read 2008). Thus, in some cases, AMF colonization may reduce invasive plant performance due to the high carbon cost of the symbiosis (Shah et al. 2009; Pringle et al. 2009). It was found that AMF had positive, neutral, and negative effects on height, biomass, and reproductive traits, respectively, of *Euphorbia dentata* (Grilli et al. 2014). Moreover, Funatsu et al. (2005) showed that the presence of AMF caused growth reduction of *Oenothera laciniata* roots.

Most previous studies concerning interactions between mycorrhizal fungi and invasive plants focused on comparing the differences in alien plant performance in the presence or absence of AMF using multi-species soil inocula. However, as it was pointed out by Stampe and Daehler (2003), the response of invaders to particular AMF species needs to be elucidated. If the degree of invasive plant response differs between AMF species, the presence of particular fungal species in a habitat may influence invasive plant performance and, as a consequence, mediate the competitive interactions between native and invasive plants (Stampe and Daehler 2003).

In Europe, one of the most important groups of alien plants is Asteraceae, which include 692 non-indigenous species (Pyšek et al. 2009). Some representatives of this family are reported as invasive (Tokarska-Guzik et al. 2012). Among them, we can distinguish a transformer category subset (Protopopova et al. 2014) that includes species changing the character, condition, form, or nature of ecosystems (Richardson et al. 2000; Tokarska-Guzik et al. 2012). According to literature (Wang and Qiu 2006; Štajerová et al. 2009) and our own observations (Majewska et al. 2015; Zubek et al. 2016), the transformers from Asteraceae are usually highly mycorrhizal. However, no studies have focused so far on the impact of AMF on these plants. Two perennial herbs of this category of invasive plants, *Rudbeckia laciniata* L. and *Solidago gigantea* Aiton, were thus chosen to determine if AMF influence their performance. We tested under laboratory conditions their response to inoculation with three commonly occurring worldwide AMF species in soils representative of two habitats invaded by *R. laciniata* and *S. gigantea*, namely fallow and river valley. The specific questions addressed in the present study included the following: (1) Are the studied plants dependent on mycorrhizal fungi for their performance? (2) To what extent AMF species affect plant mass, photosynthetic parameters, and P concentrations in biomass in two different soil types? (3) What is the relationship between the degree of mycorrhizal colonization and plant variables? Given the existence of functional diversity in arbuscular mycorrhiza (AM) symbiosis (Helgason et al. 2002; Smith and Read 2008), we hypothesized that the effects of inoculation differ between plant species, AMF species, and types of soil.

## Materials and methods

### Soils

In the experiment, we used soils collected from two sites in southern Poland, namely Kliny (49° 59′ 49.5″ N/19° 52′ 13.6″ E) and Zator (49° 59′ 59″ N/19° 26′ 40.5″ E), which represent two habitats: fallow and river valley, respectively. The selection of these habitats was performed due to the observations that fallows and river valleys are often colonized by *R. laciniata* and *S. gigantea* (Tokarska-Guzik 2005; Tokarska-Guzik et al. 2012; Domaradzki et al. 2013; Stefanowicz et al. 2016a, b; Zubek et al. 2016). These plants are able to form dense, near-monospecific patches there. The soils were transported to the laboratory in plastic containers and air-dried at room temperature. After drying, the soils were passed through 2-mm mesh to discard organic residues and then tested for physicochemical properties as detailed in Stefanowicz et al. (2016b). The physicochemical properties of the two soils are presented in Table 1. The soils were autoclaved twice at 121 °C for 1 h with a 1-week interval.

**Table 1:** The physicochemical properties of the fallow and river valley soils used in the experiment

Soil properties	Soil type	
	Fallow	River valley
Soil texture		
Sand (%)	87	82
Silt (%)	6	4
Clay (%)	7	14
Moisture	16	5
pH	6.1	8.6
Total content		
Organic C (%)	1.8	0.2
Organic C/total N	12	4
Organic C/total P	100	12
Organic C/total S	55	5
N (%)	0.15	0.05
S (%)	0.03	0.05
P (mg kg^−1^)	182	168
K (mg kg^−1^)	514	1942
Na (mg kg^−1^)	25	137
Mg (mg kg^−1^)	1165	3372
Zn (mg kg^−1^)	46	44
Ca (mg kg^−1^)	1019	5133
Extractable content (mg kg^−1^)		
P (Olsen)	1.3	6.4
Ca	604.7	559.1
K	22	66
Mg	64.4	43.1
N-NH_4_^+^	2	1
N-NO_3_^−^	10	3

### Fungi

Three common AMF species that are widely distributed throughout the world (Błaszkowski 2012) were used in the experiment: (1) *Rhizophagus irregularis* (Błaszk., Wubet, Renker & Buscot) C. Walker & A. Schüßler (=*Glomus irregulare* Błaszk., Wubet, Renker & Buscot) BEG144, (2) *Funneliformis mosseae* (T.H. Nicolson & Gerd.) C. Walker & A. Schüßler [=*Glomus mosseae* (T.H. Nicolson & Gerd.) Gerd. & Trappe] BEG12, and (3) *Claroideoglomus claroideum* IB-UJ-1 (N.C. Schenck & G.S. Sm.) C. Walker & A. Schüßler (=*Glomus claroideum* (N.C. Schenck & G.S. Sm.). Inocula of *R. irregularis*, *F. mosseae*, and *C. claroideum* were produced in 1400-ml plastic pots by adding 30 g of reference monoculture substrata of BEG 144, BEG 12, and IB-UJ-1, respectively, per pot to sterile substratum (sand + expanded garden rock + rock phosphate, 3:1:50 g/L, respectively) and planted with *Plantago lanceolata*. After 6 months, fresh AMF inocula were used in the experiment. They were composed of *P. lanceolata* roots, colonized in 60–100 % of their length, and fragments of mycelia and spores (ca. 10–15 spores per 50 g). For the control treatment, *P. lanceolata* was grown in a sterile substratum. No fungi were found in this material.

### Plants

Two invasive plant species from Asteraceae were used in the experiment: *Rudbeckia laciniata* L. and *Solidago gigantea* Aiton. *Rudbeckia laciniata*, commonly known as golden glow or cutleaf coneflower, is a perennial herb which reproduces by rhizomes and seeds (Francírková 2001). It originates from North America and was introduced to Europe as an ornamental plant. *Rudbeckia laciniata* is now invasive in this continent and penetrates into banks of rivers, streams, and ditches as well as ruderal habitats (Tokarska-Guzik 2005). *Solidago gigantea*, giant goldenrod, has the same origin (Tokarska-Guzik et al. 2012) and represents the same life form (Jacobs et al. 2004) and type of reproduction (Dajdok and Pawlaczyk 2009). This plant is highly invasive in Europe in different types of habitats (Tokarska-Guzik et al. 2012). Seeds of these species were collected in 2014 at the same sites as the soil and stored in a refrigerator for 5 months. After this time, the seeds were germinated on autoclaved, humid sand.

### Experiment setup and plant harvesting

Pots (9 cm wide, 12.5 cm high, and 500 ml in volume) were filled with 440 ml of autoclaved soils of both types. Into the center of each pot, we added 30 g of fresh inoculum, 3 cm below the surface of the soil so as to ensure the direct contact of the seedling roots with the inoculum (Janušková et al. 2013). For the control treatment, 30 g of substratum with *P. lanceolata* non-mycorrhizal roots was added. Three seedlings at the same stage of development of *R. laciniata* or *S. gigantea* were planted into each pot. After 2 weeks, two individuals were thinned out from each pot. The treatments were as follows: (1) control—soil with no AMF, (2) *Rhizophagus irregularis*, (3) *Funneliformis mosseae*, and (4) *Claroideoglomus claroideum*. In order to eliminate potential differences in bacterial community compositions between the treatments, every single pot was also filled with 4 ml of aqueous filtrate of triple inoculum mixture (20 % suspension, *w*/*v*) filtered through a Whatman no. 1 filter paper (Jansa et al. 2007), three times. For each treatment, we had 11 replicates, 176 pots in total (2 plant species × 2 types of soil × 4 treatments × 11 replicates). The pots were positioned randomly in the plant room and kept in open Sun bags (Sigma-Aldrich) to avoid contamination between treatments, at 20 ± 2 °C and the following light regime: 270–280 μmol PAR photons m^−2^ s^−1^ and 12/12 h. The plants were watered three times per week using 50 ml of distilled water.

After 3 months of *R. laciniata* and *S. gigantea* growth, we finished the experiment to assess plant performance at the early stages of their development. In order to determine plant photosynthetic performance, the measurements of chlorophyll *a* fluorescence were conducted (see below). After this procedure, the plants were harvested. They were washed in tap and then distilled water. A single plant was divided into shoots and roots. One fifth of the roots of each individual plant was cut and stained for observations of AMF structures and colonization degree assessment (see below). The shoots and roots were dried at room temperature and used for the evaluation of biomass. They were weighed using analytical balance (Radwag, WPA 60/c/1) with a precision level of 0.0001 g. The shoots and roots were also used for measurements of phosphorus concentrations (see below).

The mycorrhizal dependency (Md) of *R. laciniata* and *S. gigantea*, which is an indicator of how a plant is dependent on arbuscular mycorrhiza to produce its maximum growth, was calculated using the following equation: Md = [1 − (mean total biomass of plants without AMF / mean total biomass of plants inoculated with AMF)] × 100 % (van der Heijden 2002).

### Chlorophyll *a* fluorescence measurements

Chlorophyll *a* fluorescence was measured using a Handy PEA fluorimeter (Hansatech Instruments Ltd., King’s Lynn, Norfolk, UK). Three intact and well-developed leaves of *R. laciniata* and *S. gigantea* in each pot were dark-adapted for 30 min before measuring. The measurements were conducted according to Strasser et al. (2004) and Tsimilli-Michael and Strasser (2008). The data obtained from each individual plant were averaged. The average OJIP fluorescence transients were calculated according to the JIP test (Strasser et al. 2004) with “Biolyzer” software (Laboratory of Bioenergetics, University of Geneva, Switzerland) for each plant (sample). The performance index (PI_ABS_), which evaluates the overall photosynthetic performance (Tsimilli-Michael and Strasser 2008), was chosen for presentation.

### Determination of mycorrhizal colonization degree

The procedure of root staining for the visualization of AMF mycelia was conducted according to the Phillips and Hayman (1970) method with minor modifications (Zubek et al. 2016). Thirty 1-cm-long fragments of fine roots were randomly selected from each plant. They were mounted on slides in glycerol/lactic acid (1:1, *v*/*v*) and then squashed using cover glasses. Arbuscular mycorrhizal fungi colonization was assessed according to the Trouvelot et al. (1986) method using a Nikon Eclipse 80i light microscope with Nomarski interference contrast. The parameters analyzed were mycorrhizal frequency (*F*), relative mycorrhizal root length (*M*), and relative arbuscular richness (*A*). An estimate of *F* is given as the ratio between root fragments colonized by AMF mycelium and the total number of root fragments analyzed. The parameter *M* is an estimate of the proportion of the root cortex that is mycorrhizal relative to the whole analyzed root system. The parameter *A* is an estimate of arbuscule richness in the whole analyzed root system (Trouvelot et al. 1986).

### Measurement of phosphorus concentrations in plants

The aboveground and belowground parts of plants were dried at 80 °C and then minced with a Pulverisette 14 variable speed rotor mill (Fritsch, Germany). The concentrations of P in both shoots and roots were determined as detailed in Zubek et al. (2015).

### Statistical analysis

Two-way analysis of variance (fungal species × soil type), followed by Tukey’s (HSD) test, was performed to reveal significant differences in the mycorrhizal parameters across AMF-inoculated treatments and in photosynthetic parameters, the mass of shoots and roots, and P concentrations in the plants across all treatments, for each plant species separately. Prior to the analysis, the distribution normality was verified using the Lilliefors test. Levene’s test was performed to assess the equality of variances.

As the mycorrhizal parameters (*F*, *M*, and *A*) strongly correlated with each other (*R* > 0.9), only the relative mycorrhizal root length (*M*) was incorporated in further analyses. The correlations between the *M* parameter and the mass of shoots and roots, the photosynthetic parameter, and the P concentration in shoots and roots were tested with Pearson’s correlation coefficients separately for particular plant species, AMF inocula, and soil types. Plant parameters (*M*, plant mass, P concentration in shoots and roots) were also explored with principal component analysis (PCA) to identify the association between these traits and to recognize the grouping of samples, associated with the two different soil types and four different fungal treatments, with their similar characteristics. The analysis was based on the correlation matrix and performed for each plant species separately.

In the case of most treatments, there were 11 replicates. The exceptions were as follows: photosynthetic performance index of *R. laciniata* and P concentrations in the shoots and roots of *S. gigantea* (Table 2), where we lost single measurements due to equipment errors. The analyses were carried out using STATISTICA 10 (StatSoft, Tulsa, OK, USA) and PAST 3.10 (Hammer et al. 2001).

**Table 2:** Results of two-way ANOVA for the effects of fungal species, soil type, and their interaction on the *Rudbeckia laciniata* and *Solidago gigantea* parameters

Plant parameters		*Rudbeckia laciniata*										*Solidago gigantea*									
		Fungus			Soil			Fungus × soil			Error	Fungus			Soil			Fungus × soil			Error
		*F*	*P*	*df*	*F*	*P*	*df*	*F*	*P*	*df*	*df*	*F*	*P*	*df*	*F*	*P*	*df*	*F*	*P*	*df*	*df*
Mycorrhizal parameters	*F*—mycorrhizal frequency	*6.56*	*0.003*	2	*69.19*	*<0.001*	1	*5.11*	*0.009*	2	60	*13.29*	*<0.001*	2	*34.01*	*<0.001*	1	*4.97*	*0.01*	2	60
	*M*—relative mycorrhizal root length	*27.96*	*<0.001*	2	*136.49*	*<0.001*	1	*7.8*	*0.001*	2	60	*13.42*	*<0.001*	2	*16.73*	*<0.001*	1	1.27	0.289	2	60
	*A*—relative arbuscular richness	*26.66*	*<0.001*	2	*141.34*	*<0.001*	1	*8.13*	*0.001*	2	60	*13.23*	*<0.001*	2	*16.32*	*<0.001*	1	0.96	0.388	2	60
Shoot mass		*69.66*	*<0.001*	3	*246.06*	*<0.001*	1	*25.03*	*<0.001*	3	80	*24.64*	*<0.001*	3	*75.72*	*<0.001*	1	*6.95*	*<0.001*	3	80
Root mass		*68.86*	*<0.001*	3	*71.24*	*<0.001*	1	*8.38*	*<0.001*	3	80	*20.79*	*<0.001*	3	*44.54*	*<0.001*	1	*3.98*	*0.011*	3	80
PI_ABS_—photosynthetic performance index		0.66	0.577	3	*22.89*	*<0.001*	1	*2.76*	*0.047*	3	79	1.6	0.196	3	*31.16*	*<0.001*	1	1.9	0.136	3	80
Phosphorus concentrations in shoots		*114.38*	*<0.001*	3	0.02	0.884	1	*24.04*	*<0.001*	3	80	*12.81*	*<0.001*	3	0	0.958	1	*3.29*	*0.025*	3	77
Phosphorus concentrations in roots		*85.81*	*<0.001*	3	*20.15*	*<0.001*	1	*5.08*	*0.003*	3	80	*4.16*	*0.009*	3	0	0.953	1	*3.6*	*0.017*	3	77

The effects in italics are statistically significant

## Results

### Mycorrhizal colonization

**Fig. 1:**
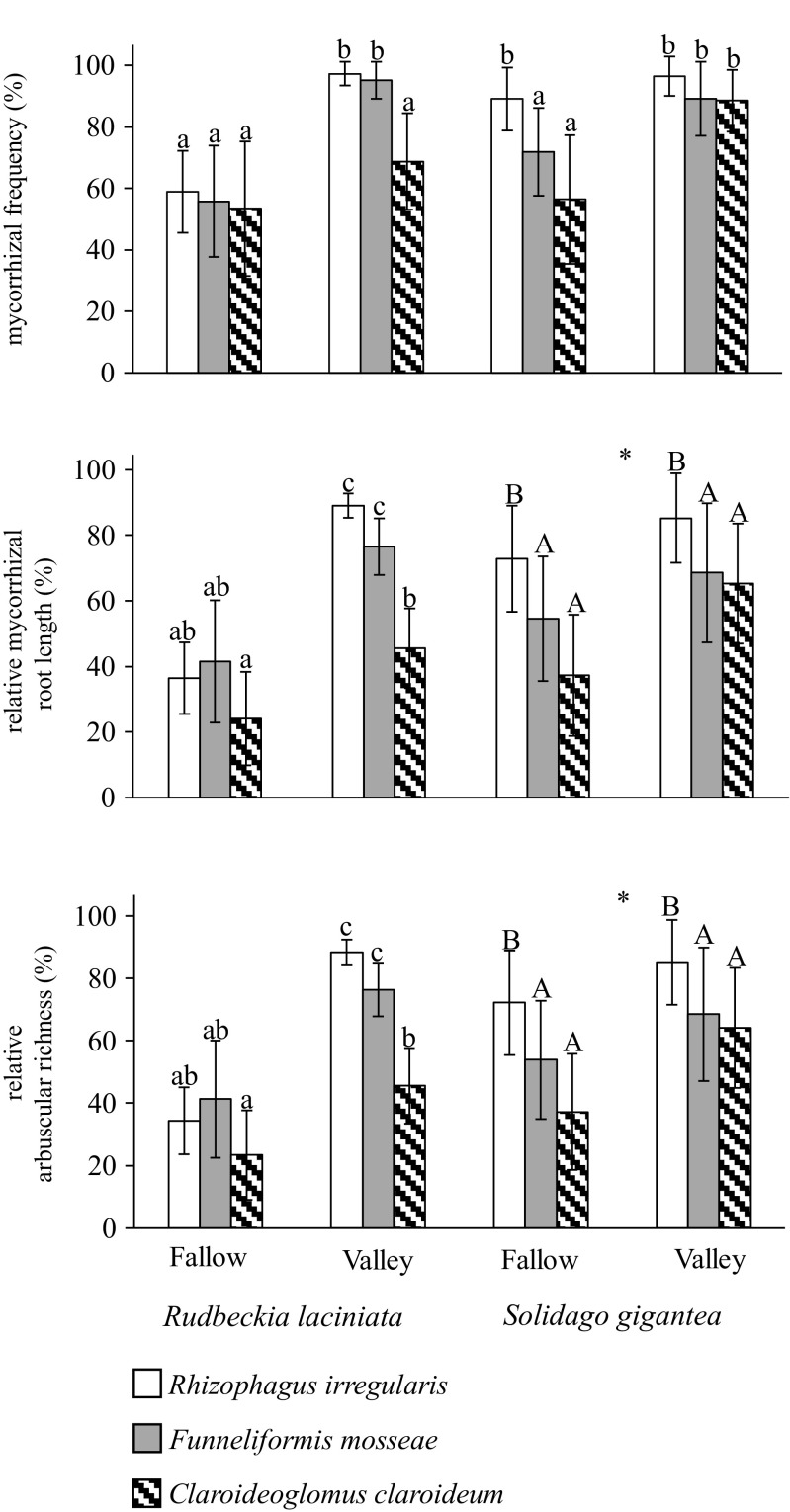
Mycorrhizal colonization (percentages; mean ± SD) of *Rudbeckia laciniata* and *Solidago gigantea* grown in the fallow and river valley soils. Mycorrhizal parameters: mycorrhizal frequency (*F*), relative mycorrhizal root length (*M*), and relative arbuscular richness (A) (see the “Materials and methods” section for a description of these parameters). Within each plant species, the *lowercase letters above the bars* indicate the statistically significant interaction between the fungus and soil effects; the *capital letters* show the significant main effect of the fungus; the *different letters above the bars* indicate statistically significant differences; the *asterisks* (*) indicate the significant main effect of the soil; for each *P* < 0.05 (see Table 2 for details on the main effects and interactions)

Arbuscular mycorrhizal fungi were found in the roots of all plants except for those of the control treatment. For *R. laciniata*, the level of AMF colonization, represented by the three mycorrhizal parameters (*F*, *M*, and *A*), was found to be higher in the case of *R. irregularis* and *F. mosseae* in river valley soil than in the other treatments. Increased levels of relative mycorrhizal root length (*M*) and relative arbuscular richness (*A*) were observed in valley soil for *C. claroideum* (significant fungus × soil interaction). In the case of *S. gigantea*, mycorrhizal frequency (*F*) was higher for both *R. irregularis* in fallow and all treatments in valley soils than in *F. mosseae* and *C. claroideum* in fallow soil (significant fungus × soil interaction). Plants growing in the soil collected within the river valley were characterized by higher values of *M* and *A* than those growing in the fallow soil (significant soil effect). Moreover, for these two parameters, *R. irregularis* was the most effective fungus in both soil types (significant fungus effect) (Table 2, Fig. 1).

### Plant growth

**Fig. 2:**
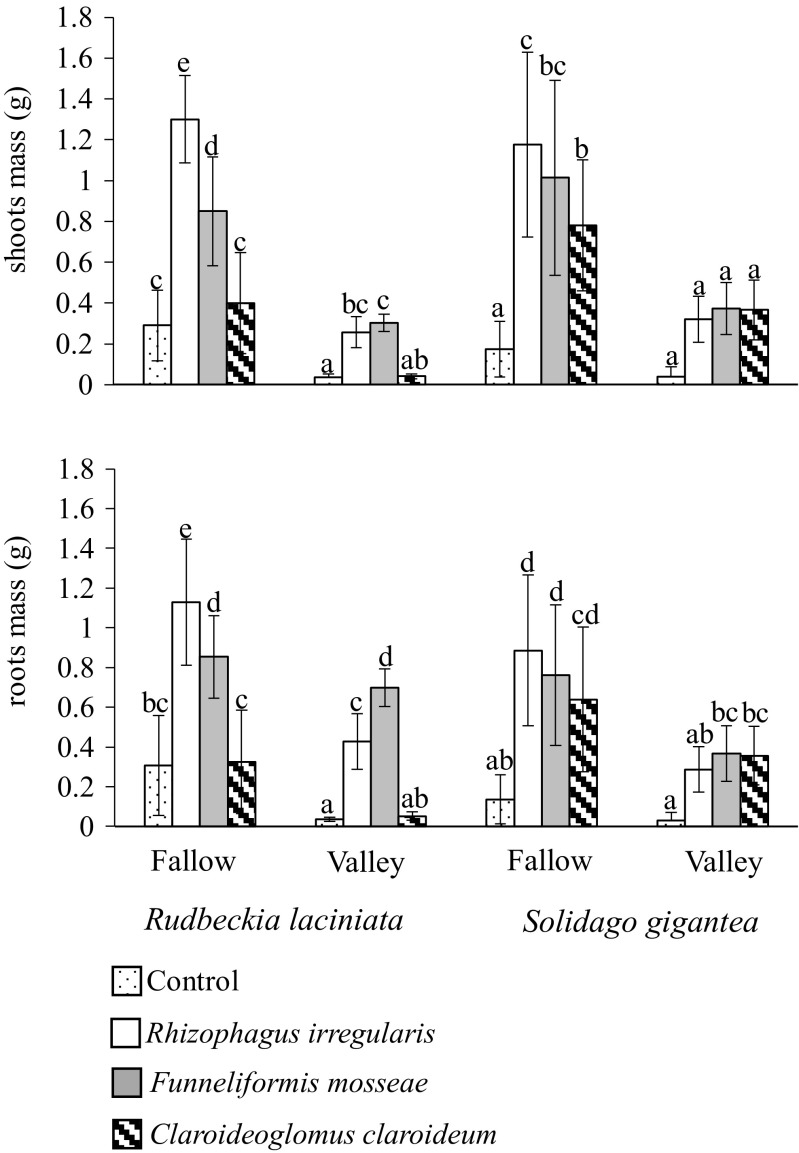
Shoot and root mass (g; mean ± SD) of *Rudbeckia laciniata* and *Solidago gigantea* grown in the fallow and river valley soils. Within each plant species, the *lowercase letters above the bars* indicate the statistically significant interaction between the fungus and soil effects; the *different letters above the bars* indicate statistically significant differences; for each *P* < 0.05 (see Table 2 for details)

In general, both invasive plant species were strongly dependent on AMF for their growth. The mycorrhizal dependency (Md) for *R. laciniata* was 88 and 63 % and for *S. gigantea* 90 and 82 % for valley and fallow soils, respectively. However, we found different effects of the fungal species in the stimulation of plant growth. *Claroideoglomus claroideum* had no impact on *R. laciniata*. Both *R. irregularis* and *F. mosseae* enhanced its mass, but their effects depended on soil type (significant fungus × soil interaction). *Rhizophagus irregularis* was more effective in the fallow soil than in the river valley soil. *Solidago gigantea* responded positively to all applied AMF species, although the differences were more visible in the fallow soil, with *R. irregularis* being most effective in increasing biomass (significant fungus × soil interaction) (Table 2, Fig. 2).

### Photosynthetic performance

**Fig. 3:**
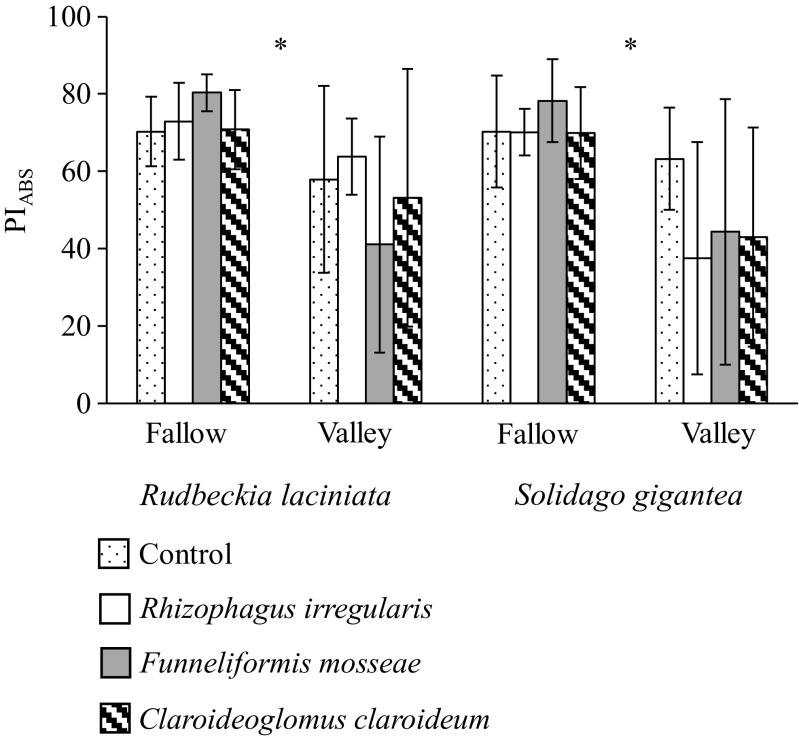
Photosynthetic performance index (PI_ABS_; mean ± SD) of *Rudbeckia laciniata* and *Solidago gigantea* grown in the fallow and river valley soils. Within each plant species, the *asterisks* (*) indicates the significant main effect of the soil (*P* < 0.05) (see Table 2 for details)

Photosynthetic performance of both plant species was influenced only by the soil type. PI_ABS_ was higher for plants growing in the fallow soil of both invasive species. Although significant fungus × soil interaction was found in the case of *R. laciniata*, the post hoc analysis did not reveal any significant differences between the treatments (Table 2, Fig. 3).

### Phosphorus concentrations in the shoots and roots

**Fig. 4:**
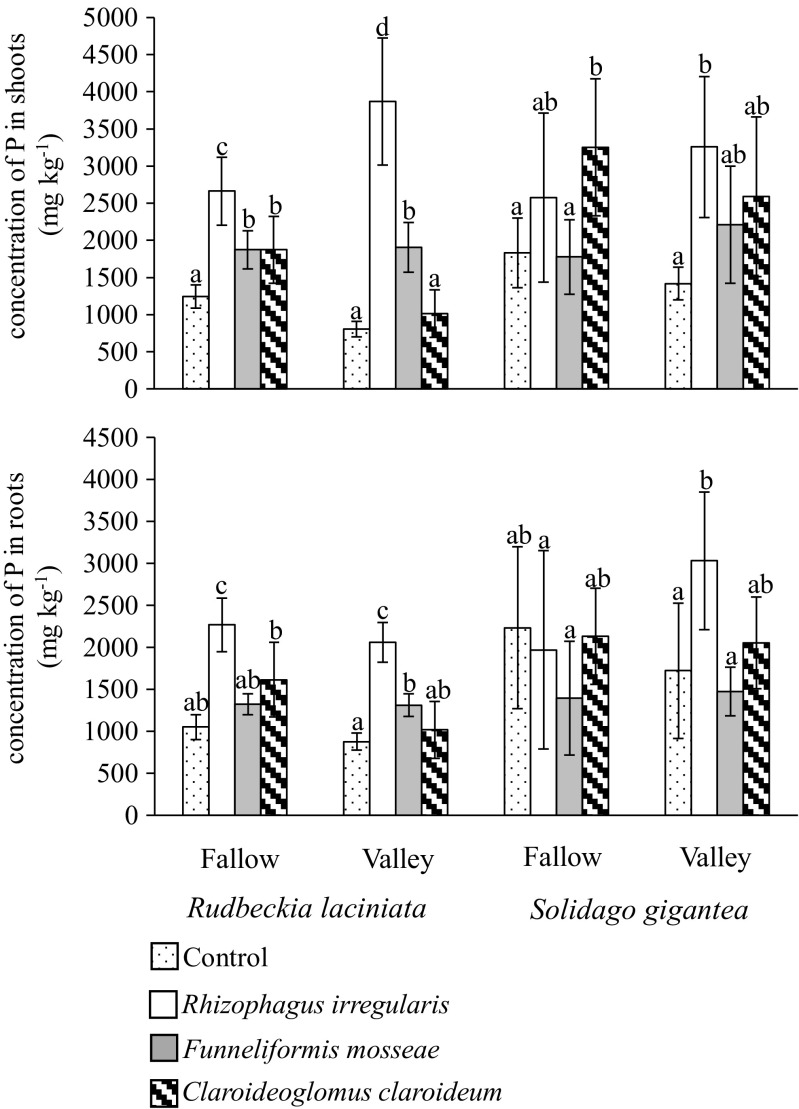
Concentrations of phosphorus (mg per kg dry weight; mean ± SD) in the shoots and roots of *Rudbeckia laciniata* and *Solidago gigantea* grown in the fallow and river valley soils. Within each plant species, the *lowercase letters above the bars* indicate the statistically significant interaction between the fungus and soil effects; the *different letters above the bars* indicate statistically significant differences; for each *P* < 0.05 (see Table 2 for details)

The concentrations of P in *R. laciniata* and *S. gigantea* mass were influenced by both AMF species and soil type (significant fungus × soil interaction, Table 2). In the case of *R. laciniata*, *R. irregularis* was most effective in the enhancement of P shoot and root concentrations. Higher concentrations of P in the shoots and roots of this plant in comparison to the control were also found after *F. mosseae* inoculation. In the case of *C. claroideum*, higher P concentrations were found only in the shoots of plants grown in the fallow soil. For *S. gigantea*, *R. irregularis* was also the most effective and its impact depended on soil type. This fungus enhanced biomass concentrations of P in the valley soil in comparison to the control. *Funneliformis mosseae* had no effect on *S. gigantea. Claroideoglomus claroideum* increased P concentration only in the shoots of plants grown in the fallow soil (Fig. 4).

### Relationships between AMF colonization degree, biomass, photosynthetic performance, and concentrations of phosphorus

The impact of mycorrhizal colonization intensity, expressed in relative mycorrhizal root length parameter (*M*), on plant mass and P concentration differed both among AMF species and between soil types. A significant correlation was found between *M* values and P concentrations in the roots of *R. laciniata* inoculated with *R. irregularis*. However, the response was the opposite in the case of the two soil types; the higher relative mycorrhizal root length of plants grown in the fallow soil resulted in higher P concentration in the roots, whereas for plants grown in the valley soil, the correlation was negative. *Claroideoglomus claroideum* showed a significant negative effect of increased mycorrhizal colonization rate of *R. laciniata* on the PI_ABS_ parameter, but only in the fallow soil. The remaining relationships for both plant species were insignificant, but in most cases, the response was different for the two types of soil within a single AMF species (Table 3).

**Table 3:** Pearson’s correlation coefficients between relative mycorrhizal root length and the mass of shoots and roots, the photosynthetic performance index, and the concentration of phosphorus in *Rudbeckia laciniata* and *Solidago gigantea* for particular arbuscular mycorrhizal fungi species treatments and soil types

Plant parameters	*Rudbeckia laciniata*						*Solidago gigantea*					
	*Rhizophagus irregularis*		*Funneliformis mosseae*		*Claroideoglomus claroideum*		*Rhizophagus irregularis*		*Funneliformis mosseae*		*Claroideoglomus claroideum*	
	Fallow	Valley	Fallow	Valley	Fallow	Valley	Fallow	Valley	Fallow	Valley	Fallow	Valley
Shoot mass	0.10	0.36	−0.17	0.26	−0.26	0.55	−0.19	0.53	−0.29	−0.15	−0.58	0.06
Root mass	−0.35	0.60	−0.18	0.53	−0.22	0.51	−0.41	0.55	−0.27	0.30	−0.54	−0.08
Phosphorus concentrations in shoots	−0.11	−0.19	−0.11	0.50	−0.21	−0.18	0.14	−0.14	0.30	−0.14	0.04	0.28
Phosphorus concentrations in roots	*0.70*	−*0.80*	−0.21	−0.22	−0.08	0.30	0.13	−0.32	0.26	−0.11	0.45	0.25
PI_ABS_—photosynthetic performance index	−0.40	−0.36	0.13	0.25	−*0.68*	0.20	−0.23	−0.32	−0.52	0.05	−0.23	0.23

Significant correlations (*P* < 0.05) are shown in italics

**Fig. 5:**
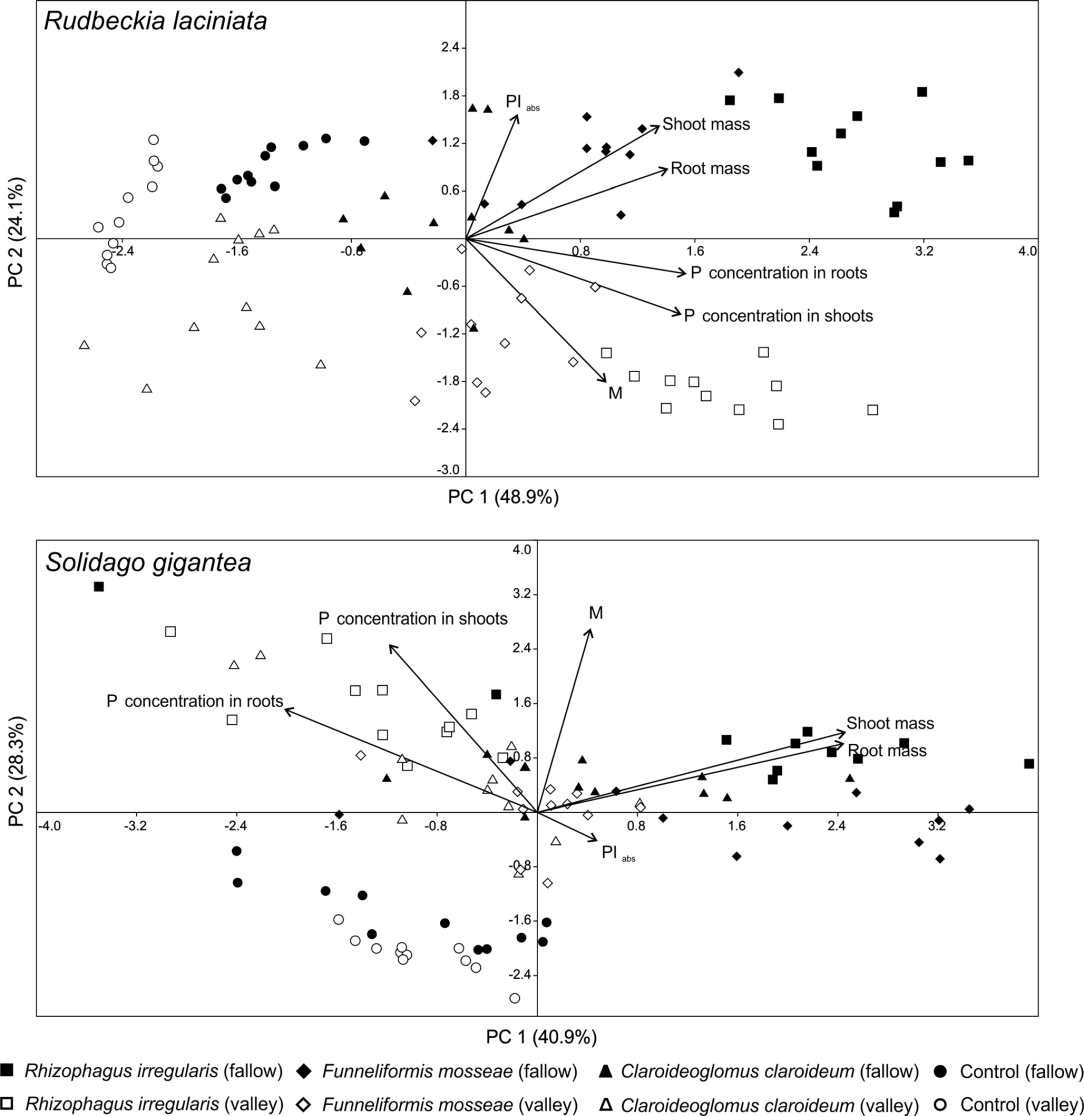
Principal component analysis ordination diagram (PC 1 vs. PC 2) of *Rudbeckia laciniata* and *Solidago gigantea* parameters (*M*—relative mycorrhizal root length, shoot and root mass, P concentration in the shoots and roots, PI_ABS_ - photosynthetic performance index) for samples of the two soil types and four fungal treatments. The percentage of total variance as explained by each axis is shown

The principal component analysis (PCA) revealed patterns in the plant parameters across all samples (Fig. 5). In the case of *R. laciniata*, PCA axis 1 was most influenced by P concentration in the shoots and roots, whereas PCA axis 2 by PI_ABS_. The scatterplot showed slightly overlapping groups of samples corresponding to particular treatments of the two soil types. The sample differentiation pattern in respect of the plant parameters is pronounced both in terms of fungal treatment and soil types. The most symptomatic differences can be observed between plant samples of *R. irregularis* in two different soil types. In the case of individuals grown in the valley soil, increased *M* rates correspond to enhanced P concentration in the shoots and roots, whereas plants harvested from the fallow soil were characterized by lower *M* values and increased biomass. In the case of *S. gigantea*, the first principal component had a high positive loading for plant mass as well as a high negative loading for P concentration in the roots. With the exception of one plant sample of *R. irregularis* harvested from the fallow soil, a general trend for fungal treatments can be observed: plants in the valley soil, grouped on the upper left side of the diagram, had higher P concentrations, whereas those in the fallow soil are characterized by increased biomass (Fig. 5).

## Discussion

We report for the first time that two transformer invasive plants from Asteraceae, *R. laciniata* and *S. gigantea*, exhibited a strong, positive growth response to AMF. Both invaders were able to grow without mycorrhizal fungi; however, in the presence of AMF, their biomass was significantly increased. More studies are needed to reveal if the advantage of higher biomass production during the early stages of growth due to AMF presence may contribute to the competitive ability of these species in colonizing new areas over the resident plants. Although Bunn et al. (2015) did not support this hypothesis in their meta-analysis, it is difficult to generalize on the AMF effects on invasive plants. As shown in this and other studies, the symbiotic interaction depends on the identity of plant and AMF species, the degree of mycorrhizal colonization, and the soil properties (Smith and Read 2008).

In line with our expectations, different effects of the fungal species were observed in the case of their impact on both plants, with *R. irregularis* and *C. claroideum* being the most and least effective symbionts, respectively. Bray et al. (2003) found that only a mix of AMF isolated from the roots of *Ardisia crenata*, but not the laboratory-available inocula containing *Claroideoglomus etunicatum* or *Rhizophagus fasciculatus*, improved seedling growth of this invasive plant. The study by Shah et al. (2008a) confirmed the reliance of *Anthemis cotula* growth on mycorrhizal fungi with more favorable effects of resident, from invaded habitat, than foreign AMF strains. The different effects of AMF species were also found for *Solidago canadensis* (Zhang et al. 2010; Yuan et al. 2014). *Rudbeckia laciniata* and *S. gigantea* tended to increase AMF abundance in the field (Zubek et al. 2016). This finding and the observations from our present study support the enhanced mutualism hypothesis for these invasive species; their invasion may be facilitated by AMF with strong beneficial effects (Reinhart and Callaway 2006; Bunn et al. 2015).

*Claroideoglomus claroideum* and *F. mosseae* are among the most widespread AMF in disturbed habitats in Europe, such as agricultural areas (Oehl et al. 2003, 2004; Vestberg et al. 2005; Zubek et al. 2012, 2013) and river valleys (Nobis et al. 2015). These two species were also most frequently found in the locations where *R. laciniata* and *S. gigantea* form monospecific patches. However, we did not detect *R. irregularis* in soils under either invader (Zubek et al. 2016). This species is widely distributed throughout the world, although rather rarely recorded probably because of the low production of extraradical spores (Błaszkowski 2012; Błaszkowski et al. 2008). Thus, the absence of *R. irregularis* under *R. laciniata* and *S. gigantea* could be due to the method applied, as the study was based on the identification of spores isolated directly from soils (Zubek et al. 2016). The identity of AMF species colonizing *R. laciniata* and *S. gigantea* in the field remains to be investigated using molecular tools. Nevertheless, our observations from this study suggest that the differences in the AMF species composition in the aforementioned habitats may differentially influence growth and phosphorus content of these invasive plants. Stampe and Daehler (2003) showed that the composition of the AMF community can affect plant community and invasion success.

In addition to the AMF species identity, other important factors affecting AMF-plant interactions include the degree of mycorrhizal colonization and the soil properties. All these factors are interrelated, because physicochemical soil parameters influence the development and functioning of AM (Smith and Read 2008). The soils of the two habitats invaded by *R. laciniata* and *S. gigantea* that were applied in our study differed in several physicochemical properties. The fallow soil was moderately acidic and had higher amounts of nutrients necessary for basic plant nutrition, such as total and extractable nitrogen and total phosphorus. It also contained more organic carbon. The river valley soil was characterized by alkaline pH and a higher amount of available P as well as secondary macronutrients (Ca, S, Mg). Firstly, the higher degree of mycorrhizal colonization of plants harvested from the valley soil may be associated with soil pH. Several studies showed that in the case of various plant species, root colonization by AMF was stimulated by increased soil alkalinity (Postma et al. 2007; Zubek et al. 2009; Ouzounidou et al. 2015). In the case of the invasive plant *Impatiens parviflora*, AM colonization degree also increased along with increasing soil pH (Chmura and Gucwa-Przepióra 2012). Secondly, soil fertility is considered an important factor affecting mycorrhizal association. Generally, soils low in mineral nutrients limit plant development and increase the dependence of plants on mycorrhiza (Siqueira and Saggin Júnior 1995). Lett et al. (2011) found that under low P conditions, mycorrhiza appeared to be beneficial to the growth of the invasive liana, *Celastrus orbiculatus*, and this could be related to the invasion success of this plant. Although no clear trends can be seen in the fertility of the soils applied in our experiment, higher mycorrhizal dependency of both plants was observed in the valley soil.

In some cases, high mycorrhizal colonization rate can negatively affect plant growth or can decrease plant mass due to the carbon costs for the maintenance of a fungal symbiont (Smith and Smith 2011a, b, 2012). Nevertheless, Treseder (2013) in a meta-analysis showed that plants with a greater percent of root length colonized received more phosphorus from AMF, which usually led to increased plant growth and, consequently, biomass, albeit with variability between fungal and plant species as well as environmental conditions. Bray et al. (2003) found that *Ardisia crenata* had a differential response to different inocula applied. In one case, higher mycorrhizal colonization rates resulted in higher growth and P content of this invader. For other AMF species, *A. crenata* colonization degree was not related to P concentration, and the lack of positive effects on growth was observed despite the fact that these fungi enhanced tissue P concentration. Harner et al. (2010) reported a positive relationship between the degree of AMF colonization and biomass of *Centaurea stoebe*. In our study, the effect of mycorrhizal colonization rate on plant mass and P concentration was diversified both among AMF species and between types of soils. The most distinct and repeatable response was found in the case of *R. laciniata* inoculated with *R. irregularis*. The plants grown in the fallow soil were characterized by a lower mycorrhizal colonization rate, and the positive effect of AMF on plant mass was, in this case, noticeable. On the contrary, in the valley soil, the positive effect of this fungus on P concentration was more pronounced than in the fallow soil, and this impact was more powerful with increasing mycorrhizal colonization rate. It is possible that increased C allocation into the fungus resulted in lower biomass but was rewarded with enhanced P concentration. Nevertheless, *R. laciniata* individuals colonized by *R. irregularis* had always significantly higher shoot and root mass than those of the non-mycorrhizal control irrespective of the soil type. The other AMF species showed various responses with respect to relations between the mycorrhizal colonization level and plant mass as well as P concentration, but in most cases, the response for a single AMF species was different in the two soil types. For *S. gigantea*, the relationships between mycorrhizal colonization rate and plant mass as well as P concentration were less apparent. However, a general trend within fungal treatments was noticeable, where plants harvested from fallow soil were mainly associated with increased biomass.

## Conclusions

Our investigation included three common AMF species and soils representative of two habitats, thus enabling strong inferences on the effect of mycorrhizal fungi on *R. laciniata* and *S. gigantea*. We report here for the first time that the growth and P content of these invaders of the transformer category are enhanced by AMF. The direction and magnitude of the changes in both plant species due to AMF were dependent on the fungal and soil identities. Therefore, the presence of particular AMF species in a site may determine the invasive plant’s success. Our findings indicate that AMF have a direct effect on the early stages of *R. laciniata* and *S. gigantea* growth. Further studies, however, are required to compare the effects of AMF, also with using different combinations of AMF strains autochthonous to the tested soils, on both invasive and co-occurring native plants, to determine if the enhanced growth and P concentration of *R. laciniata* and *S. gigantea* due to AMF provide a competitive advantage over resident species and permit these invaders to gain dominance.
